# Subcutaneous abatacept for the treatment of rheumatoid arthritis in routine clinical practice in Germany, Austria, and Switzerland: 2-year retention and efficacy by treatment line and serostatus

**DOI:** 10.1007/s10067-023-06649-x

**Published:** 2023-06-14

**Authors:** Rieke Alten, Hans-Peter Tony, Bettina Bannert, Hubert Nüßlein, Christiane Rauch, Sean E. Connolly, Melanie Chartier, Karissa Lozenski, Roland Hackl, Adrian Forster, Peter Peichl

**Affiliations:** 1 Department of Internal Medicine, Rheumatology, Schlosspark-Klinik, University Medicine Berlin, Heubnerweg 2, 14059 Berlin, Germany; 2 Medizinische Klinik Und Poliklinik II, Rheumatologie/Klinische Immunologie, Universitätsklinikum Würzburg, Josef-Schneider-Straße 2, 97080 Würzburg, Germany; 3 Rheumatologische Universitätsklinik, Universitätsspital Basel, Petersgraben 4, 4031 Basel, Switzerland; 4 Medic-Center Nürnberg (Private Practice), Gibitzenhofstraße 150, 90443 Nuremberg, Germany; 5 Medical Immunology & Fibrosis, Bristol Myers Squibb, Arnulfstraße 29, 80636 Munich, Germany; 6 Immunology and Fibrosis/Global Drug Development, Bristol Myers Squibb, 3401 Princeton Pike, NJ 08540 Lawrenceville, USA; 7 MESP France – Market Access, Bristol Myers Squibb, 3 Rue Joseph Monier, 92506 Rueil-Malmaison, France; 8 Immuno-Oncology, Bristol Myers Squibb, Handelskai 92/Rivergate/Gate 1, 5. OG, 1200 Vienna, Austria; 9 Department of Rheumatology, Schulthess Klinik, Lengghalde 2, 8008 Zurich, Switzerland; 10 Department of Internal Medicine, Evangelical Hospital, Hans-Sachs-Gasse 10-12, 1180 Vienna, Austria

**Keywords:** Abatacept, bDMARD, Clinical response, Retention, Rheumatoid arthritis, Serostatus

## Abstract

**Introduction/objectives:**

The ASCORE study on treatment for rheumatoid arthritis (RA) showed better retention and clinical response rates for abatacept as first-line versus later-line therapy. This post hoc analysis of ASCORE assessed 2-year retention, efficacy, and safety of subcutaneous (SC) abatacept in Germany, Austria, and Switzerland.

**Methods:**

Adults with RA who initiated SC abatacept 125 mg once weekly were assessed. Primary endpoint was abatacept retention rate at 2 years. Secondary endpoints: proportions of patients with low disease activity (LDA)/remission per Disease Activity Score in 28 joints based on erythrocyte sedimentation rate (≤ 3.2), Simplified Disease Activity Index (≤ 11), and Clinical Disease Activity Index (≤ 10). Outcomes were analyzed by treatment line and serostatus.

**Results:**

For the pooled cohort, the 2-year abatacept retention rate was 47.6%; retention was highest in biologic-naïve patients (50.5% [95% confidence interval 44.9, 55.9]). Patients seropositive for both anti-citrullinated protein antibody (ACPA) and rheumatoid factor (RF; + / +) at baseline had a higher 2-year abatacept retention rate than patients with single seropositivity for either APCA or RF or double-seronegativity (− / −), irrespective of treatment line. At 2 years, a higher proportion of patients who were biologic-naïve were in LDA/remission than patients with one or ≥ two prior biologics.

**Conclusion:**

A higher proportion of patients with + / + RA (compared with − / − RA) had abatacept retention after 2 years. Early identification of patients with seropositive RA may facilitate a precision-medicine approach to RA treatment, leading to a higher proportion of patients in LDA/remission.

**Trial registration number:**

NCT02090556; date registered: March 18, 2014 (retrospectively registered).

**Table:** 

**Key Points** *• This post hoc analysis of a German-speaking subset of European patients with RA from the global ASCORE study (NCT02090556) showed that retention of SC abatacept within this subset was 47.6%, with good clinical outcomes after 2 years.* *• Patients with double-seropositive RA (ACPA and RF positive) had higher retention of abatacept than patients with double-seronegative RA (ACPA and RF negative). Retention and clinical responses were highest for patients who were biologic-naïve compared with patients who had one or ≥ two prior biologic treatments.* *• These real-world data may be useful for clinicians in informing individualized treatment pathways for patients with RA, and fostering superior disease control and clinical outcomes.*

**Supplementary information:**

The online version contains supplementary material available at 10.1007/s10067-023-06649-x.

## Introduction

Rheumatoid arthritis (RA) is a chronic inflammatory joint disease [1] that is complicated by the presence of rheumatoid factor (RF) and anti-citrullinated protein antibodies (ACPAs). RF and ACPAs are associated with a severe and aggressive RA disease course [2], including rapid joint destruction [3] and an increased risk of mortality [4], and are therefore included in the most recent diagnostic criteria as an indicator of poor prognosis of RA[5]. An RA diagnosis results in a high burden to patients, their families, and society, in terms of patient quality of life and economic considerations that significantly factor into disease management [6]. These considerations have together led to the endorsement of a treat-to-target approach for RA [6, 7], which is a systematic approach involving frequent monitoring of disease activity and treatment modification to minimize disease activity [6]. The goal of the treat-to-target approach is clinical remission or, alternatively, low disease activity (LDA) [6, 7].

Conventional synthetic disease-modifying antirheumatic drugs (csDMARDs), such as methotrexate, are currently recommended as the first-line treatment for patients with RA [6, 7]. The American College of Rheumatology (ACR) and the European Alliance of Associations for Rheumatology (EULAR) recommend early use of immunomodulatory biologic (b) and targeted synthetic DMARDs when csDMARDs fail to reach the therapeutic target by 3 to 6 months [6, 7], particularly when factors indicative of poor prognosis are evident, such as the presence of RF and/or ACPA.

Abatacept, a bDMARD, is a selective co-stimulation modulator that blocks the interaction between CD80/CD86 on antigen-presenting cells and CD28 on T cells, disrupting T-cell activation [8]. Abatacept is approved for the treatment of moderate-to-severe RA and is available in both intravenous (IV) and subcutaneous (SC) formulations [9]. Depending on location, abatacept is authorized as a monotherapy or concomitantly with other DMARDs such as methotrexate and has proven efficacy and safety for the treatment of patients with RA [10–13]. Long-term efficacy and safety are comparable between SC and IV abatacept [14]; however, the SC formulation has the advantage of providing a more convenient and flexible route of administration with the option for self-administration, and is associated with decreased costs [14, 15]. Additionally, patients with double-seropositive RA (ACPA + and RF + ; + / +) treated with abatacept experienced greater improvements in clinical outcomes and fewer discontinuations than those with double-seronegative RA (ACPA − and RF − ; − / −) [16].

The Abatacept SubCutaneOus in Routine clinical practicE (ASCORE; ClinicalTrials.gov: NCT02090556) study was a 2-year, observational, prospective, multicenter study of SC abatacept for the treatment of patients with moderate-to-severe active RA in routine clinical practice. Patients were included from 11 countries (Austria, Australia, France, Monaco, Germany, Greece, Italy, Netherlands, Spain, Switzerland, and UK) [17]. Of these, patients from German-speaking countries (Germany, Austria, and Switzerland) made up the largest patient group in the overall ASCORE study population, accounting for a third.

Interim analyses of the ASCORE study reported better retention and clinical response rates when SC abatacept was administered as a first-line bDMARD rather than a later-line bDMARD [18, 19]. At 2 years, SC abatacept retention was 47% (irrespective of treatment line) with good clinical outcomes and no new safety signals [17]. Notably, retention and clinical response rates at 2 years were higher in patients who received abatacept as an early-line bDMARD than in patients who received it as a later-line bDMARD [17]. Abatacept retention rates were also higher for patients with + / + RA than for patients with − / − RA [17].

Observational studies capture data from patients who may otherwise have not been selected for clinical trials due to stringent selection criteria, or from patients belonging to vulnerable populations. As such, real-world data can provide healthcare professionals with enhanced and valuable insights on the real-life long-term management and outcomes of patients with RA. Additionally, local breakouts of populations within a global study may provide important and relevant guidance for local physicians. Thus, this post hoc analysis of the ASCORE study aimed to investigate the retention, efficacy, and safety of SC abatacept by treatment line and serostatus in routine clinical practice in Germany, Austria, and Switzerland after 2 years.

## Materials and methods

### Study design

Patients were recruited for the ASCORE study (ClinicalTrials.gov: NCT02090556) from February 2013 to April 2017 from 11 countries (Austria, Australia, France, Monaco, Germany, Greece, Italy, Netherlands, Spain, Switzerland, and UK). All participating countries were required to have regulatory approval and a reimbursement policy for abatacept to ensure availability of the drug to all eligible patients. SC abatacept was initiated under the guidance of a physician and in accordance with local routine practices [11]. Rheumatologists were randomly selected for a well-balanced geographic distribution and were representative of specialists caring for patients with RA in each participating country.

Patients who met the inclusion criteria were followed up approximately every 3 months for 30 months in line with routine clinical practice. Patients who discontinued SC abatacept, regardless of the reason and time of discontinuation, were followed up to the planned 24-month follow-up.

### Study population

Patients aged ≥ 18 years with moderate-to-severe RA (as defined by the ACR/EULAR 2010 criteria [5]) who initiated SC abatacept (125 mg weekly) were enrolled into three cohorts: biologic-naïve, one prior biologic treatment, and ≥ two prior biologic treatments. Patients who were participating in any interventional clinical trial in RA at the time were excluded.

The study was conducted in accordance with the 1964 Declaration of Helsinki and its later amendments, the International Conference on Harmonization Good Clinical Practice Guidelines, and the International Society for Pharmacoepidemiology (ISPE) Guidelines for Good Epidemiology Practices.

The laws and regulatory requirements of all countries participating in this study were adhered to. The study protocol and patient enrollment materials were approved according to local law in each participating country prior to initiation of the study.

All participants signed an informed consent form prior to their participation in the study and agreed to have their data published for research purposes, given that the data provided were first anonymized.

### Post hoc analysis endpoints and assessments

This post hoc analysis included patients from Germany, Austria, and Switzerland — the German-speaking countries included in the ASCORE study. Patient demographics and disease characteristics were recorded by treatment line and country (data for patients in Switzerland with ≥ two prior biologics are not presented due to a small sample size).

The proportion of patients with SC abatacept retention over 2 years was analyzed by treatment line and country; abatacept retention was defined as consecutive time on treatment over 2 years. The proportion of patients with clinical outcomes of LDA/remission according to Disease Activity Score in 28 joints based on the erythrocyte sedimentation rate (DAS28 [ESR]; ≥ 2.6 to ≤ 3.2 and < 2.6, respectively), Simplified Disease Activity Index (SDAI; > 3.3 to ≤ 11 and ≤ 3.3, respectively), and Clinical Disease Activity Index (CDAI; > 2.8 to ≤ 10 and ≤ 2.8, respectively) were also assessed by treatment line [20] for the pooled and German cohorts (data are not presented for Austria and Switzerland due to small sample sizes).

Further analyses included assessing patient demographics and disease characteristics, and the proportion of patients with SC abatacept retention over 2 years, by treatment line and serostatus for the pooled and German cohorts (data are not presented for Austria and Switzerland, or for patients with one prior biologic treatment, due to small sample sizes).

Safety was monitored and evaluated in accordance with local regulations. The drug manufacturer’s pharmacovigilance department was notified of any adverse events (AEs) or serious AEs (SAEs) noted by the treating physician, irrespective of whether they were deemed related to abatacept. An SAE was defined as an AE that was fatal or life-threatening, required extended hospitalization, led to persistent or significant disability or incapacity, induced a birth defect, or was considered an important medical event. The number of deaths was also recorded. Safety data for patients in Switzerland with ≥ two prior biologics are not presented due to a small sample size.

### Statistical analyses

Data were analyzed as a pooled data set (encompassing all patients included in the Germany, Austria, and Switzerland cohorts) and were stratified by treatment line as biologic-naïve, one prior biologic, and ≥ two prior biologics. Serostatus was defined as + / + , single-positive (ACPA + or RF + ; + / −), and − / − RA. Baseline demographics and disease characteristics were analyzed descriptively and reported as mean (standard deviation [SD]) for continuous variables and *n* (%) for categorical variables. The number and reasons for abatacept discontinuations were recorded by rheumatologists. Reasons were recorded in an open-ended text box for the category “other”. Adjusted risk (hazard ratio, 95% confidence intervals [CIs]) of treatment discontinuation according to treatment and baseline serostatus was calculated using a Cox proportional hazards model. Retention of abatacept was estimated using Kaplan–Meier analysis with 95% CIs for comparison of patients stratified by treatment line. Clinical outcomes at 2 years were reported as percentages. Safety was analyzed descriptively throughout the study.

## Results

### Patient disposition, demographics, and disease characteristics

**Fig. 1:**
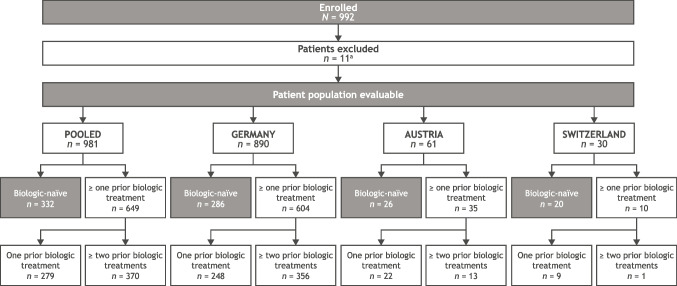
Patient disposition for post hoc analysis population. ^a^All exclusions were from the Germany cohort: prior abatacept use/not initiated with subcutaneous abatacept, *n* = 9; age/sex missing, *n* = 1; abatacept intake date missing, *n* = 1

Overall, 992 (34.3%) patients from ASCORE’s total evaluable study population of 2892 were included in this post hoc analysis (Fig. 1). Of these, 11 patients were excluded due to: prior abatacept use/not initiated with SC abatacept (*n* = 9), missing age/sex (*n* = 1), or missing abatacept intake date (*n* = 1), leaving 981 patients included in the pooled evaluable patient cohort (Fig. 1). Of these, 890 were from Germany, 61 were from Austria, and 30 were from Switzerland. In total, 332 (33.8%) patients were biologic-naïve, 279 (28.4%) received one prior biologic, and 370 (37.7%) received ≥ two prior biologics (Fig. 1).

Patient demographics and baseline disease characteristics were generally similar across treatment lines (Table 1). The mean (SD) age of patients was 58.2 (12.8) years, 756 (77.1%) patients were female, 456 (69.0%) were ACPA positive, and 481 (64.6%) were RF positive. Both radiographic erosion and Health Assessment Questionnaire-Disability Index (HAQ-DI) values were higher in the ≥ two prior biologics group than in the biologic-naïve or one prior biologic groups. The proportion of patients with radiographic erosion ranged from 50.6% in patients who were biologic-naïve to 65.1% in patients with ≥ two prior biologics, and the HAQ-DI score ranged from 1.1 in patients who were biologic-naïve to 1.4 in patients with ≥ two prior biologics. RA duration was more varied across treatment lines, ranging from 9.1 years in the biologic-naïve group to 14.9 years in the ≥ two prior biologics group. Overall, > 70% of patients were co-prescribed corticosteroids or methotrexate with abatacept.

**Table 1:** Patient demographics and baseline disease characteristics, overall and by treatment line

	Overall	Pooled			Germany			Austria			Switzerland^a^	
			Prior biologic			Prior biologic			Prior biologic			Prior biologic
	*N* = 981	Biologic-naïve, *n* = 332	One, *n* = 279	≥ two, *n* = 370	Biologic-naïve, *n* = 286	One, *n* = 248	≥ two, *n* = 356	Biologic-naïve, *n* = 26	One, *n* = 22	≥ two, *n* = 13	Biologic-naïve, *n* = 20	One, *n* = 9
Characteristic												
Age, years	58.2 (12.8)	59.0 (13.6)	58.2 (12.8)	57.6 (12.1)	59.2 (13.6)	58.6 (12.6)	57.6 (12.0)	58.0 (14.2)	54.6 (12.8)	55.1 (13.3)	58.2 (13.4)	55.9 (18.1)
Sex, female, *n* (%)	756 (77.1)	243 (73.2)	220 (78.9)	293 (79.2)	206 (72.0)	189 (76.2)	282 (79.2)	19 (73.1)	22 (100.0)	11 (84.6)	18 (90.0)	9 (100.0)
BMI, kg/m^2^	27.5 (5.7) *n* = 963	27.2 (5.6) *n* = 327	28.1 (5.9) *n* = 274	27.3 (5.7) *n* = 362	27.5 (5.6) *n* = 282	28.3 (5.8) *n* = 245	27.4 (5.8) *n* = 348	27.1 (6.0) *n* = 25	26.5 (5.5) *n* = 21	25.9 (5.3)	23.9 (4.1)	29.0 (9.1) *n* = 8
RA duration, years	12.1 (9.6) *n* = 973	9.1 (8.9) *n* = 328	11.8 (9.0) *n* = 277	14.9 (9.8) *n* = 368	9.6 (8.5) *n* = 282	12.2 (9.1) *n* = 246	15.2 (9.9) *n* = 354	5.4 (7.1)	9.5 (8.1)	9.2 (6.2)	7.6 (14.2)	4.8 (4.1)
Presence of radiographic erosion, *n* (%)	384 (57.5) *n* = 668	118 (50.6) *n* = 233	102 (55.7) *n* = 183	164 (65.1) *n* = 252	100 (52.1) *n* = 192	92 (56.8) *n* = 162	158 (65.0) *n* = 243	7 (31.8) *n* = 22	6 (46.2) *n* = 13	5 (62.5) *n* = 8	11 (57.9) *n* = 19	4 (50.0) *n* = 8
ACPA positive, *n* (%)	456 (69.0) *n* = 661	168 (68.9) *n* = 244	135 (71.8) *n* = 188	153 (66.8) *n* = 229	147 (72.8) *n* = 202	118 (73.3) *n* = 161	147 (67.1) *n* = 219	12 (50.0) *n* = 24	14 (73.7) *n* = 19	6 (66.7) *n* = 9	9 (50.0) *n* = 18	3 (37.5) *n* = 8
RF positive, *n* (%)	481 (64.6) *n* = 745	180 (66.4) *n* = 271	136 (65.1) *n* = 209	165 (62.3) *n* = 265	158 (68.7) *n* = 230	119 (65.0) *n* = 183	160 (63.2) *n* = 253	13 (54.2) *n* = 24	13 (68.4) *n* = 19	5 (45.5) *n* = 11	9 (52.9) *n* = 17	4 (57.1) *n* = 7
CRP, mg/L	14.4 (21.0) *n* = 837	15.7 (22.4) *n* = 287	13.2 (19.4) *n* = 238	14.1 (20.8) *n* = 312	16.8 (23.5) *n* = 245	14.1 (20.4) *n* = 208	13.5 (19.5) *n* = 298	10.6 (14.9) *n* = 25	6.5 (8.4)	17.2 (25.4)	5.8 (6.5) *n* = 17	9.2 (8.6) *n* = 8
ESR, mm	29.0 (22.6) *n* = 778	29.0 (20.4) *n* = 267	31.4 (24.0) *n* = 222	27.1 (23.3) *n* = 289	30.7 (20.7) *n* = 227	32.7 (24.3) *n* = 194	26.9 (23.5) *n* = 277	23.2 (17.8) *n* = 25	22.7 (17.4) *n* = 20	29.4 (21.4) *n* = 11	12.6 (9.3) *n* = 15	22.3 (28.4) *n* = 8
HAQ-DI score	1.3 (0.7) *n* = 373	1.1 (0.7) *n* = 147	1.3 (0.7) *n* = 111	1.4 (0.7) *n* = 115	1.2 (0.7) *n* = 112	1.3 (0.7) *n* = 88	1.5 (0.7) *n* = 105	1.0 (0.8) *n* = 20	1.3 (0.6) *n* = 17	1.1 (0.7) *n* = 9	0.7 (0.56) *n* = 15	0.7 (0.47) *n* = 6
Patient pain, 0–100 mm VAS	56.8 (23.0) *n* = 596	55.4 (22.7) *n* = 220	58.9 (23.4) *n* = 174	56.6 (23.0) *n* = 202	57.4 (23.2) *n* = 169	59.3 (23.1) *n* = 146	57.0 (23.7) *n* = 190	56.9 (21.0) *n* = 23	55.6 (22.0) *n* = 20	60.7 (23.3) *n* = 10	49.3 (17.9) *n* = 19	67.1 (20.3)
PGA, 0–100 mm VAS	57.8 (23.3) *n* = 583	56.7 (23.5) *n* = 209	59.9 (22.6) *n* = 173	57.0 (23.7) *n* = 201	55.9 (23.4) *n* = 178	58.8 (23.8) *n* = 145	56.2 (23.0) *n* = 191	58.3 (27.6) *n* = 23	62.9 (17.6) *n* = 18	56.1 (23.0) *n* = 10	47.6 (18.8) *n* = 17	62.8 (25.4)
Treatment patterns												
IV abatacept, *n* (%)	80 (8.2) *n* = 976	23 (6.9)	26 (9.5) *n* = 275	31 (8.4) *n* = 369	20 (7.0)	25 (10.2) *n* = 244	29 (8.2) *n* = 355	3 (11.5)	1 (4.5)	2 (15.4)	0 (0.0)	0 (0.0)
bDMARD monotherapy, *n* (%)	60 (6.1) *n* = 979	19 (5.7)	23 (8.3) *n* = 278	18 (4.9) *n* = 369	11 (3.8)	13 (5.3) *n* = 247	13 (3.7) *n* = 355	6 (23.1)	8 (36.4)	4 (30.8)	2 (10.0)	2 (22.2)
Concomitant non-bDMARDs, *n* (%)	777 (79.2)	284 (85.5)	205 (73.5)	288 (77.8)	252 (88.1)	189 (76.2)	281 (78.9)	16 (61.5)	10 (45.5)	7 (53.8)	16 (80.0)	6 (66.7)
Corticosteroids co-prescribed with abatacept, *n* (%)	715 (72.9)	236 (71.1)	196 (70.3)	283 (76.5)	214 (74.8)	184 (74.2)	278 (78.1)	11 (42.3)	8 (36.4)	5 (38.5)	11 (55.0)	4 (44.4)
MTX co-prescribed with abatacept, *n* (%)	697 (71.0)	248 (74.7)	186 (66.7)	263 (71.1)	227 (79.4)	178 (71.8)	258 (72.5)	10 (38.5)	7 (31.8)	5 (38.5)	11 (55.0)	1 (11.1)

Data are shown as mean (SD) unless otherwise specified. Where data were not available for all patients, *n* values are presented

^a^Data for Switzerland ≥ two prior biologics treatment group are not presented to maintain confidentiality due to small patient population (*n* = 1)

*ACPA*, anti-citrullinated protein antibody; *bDMARD*, biologic disease-modifying antirheumatic drug; *BMI*, body mass index; *CRP*, C-reactive protein; *ESR*, erythrocyte sedimentation rate (after 1 h); *HAQ-DI*, Health Assessment Questionnaire-Disability Index; *IV*, intravenous; *MTX*, methotrexate; *PGA*, Patient Global Assessment of disease activity; *RA*, rheumatoid arthritis; *RF*, rheumatoid factor; *SD*, standard deviation; *VAS*, visual analog scale

Generally, patient demographics and baseline disease characteristics were similar across the Germany, Austria, and Switzerland cohorts and treatment lines (Table 1).

In total, 632 patients were included in further analyses that assessed patients by treatment line and serostatus. Of these, 57.3% (*n* = 362) had + / + RA, 18.7% (*n* = 118) had + / − RA, and 24.1% (*n* = 152) had − / − RA. When stratified by baseline serostatus, patient demographics and baseline disease characteristics were similar in both the pooled and Germany cohorts (Supplementary Tables 1 and 2). Overall, for patients with serostatus recorded at baseline, 67.8% were single-seropositive for ACPA and 32.2% were single-seropositive for RF (Supplementary Table 1). Similar proportions of patients were single seropositive for either ACPA or RF at baseline in the German cohort (70.1% and 29.9%, respectively; Supplementary Table 2). Baseline disease activity measures were well-balanced across treatment lines and serostatus groups.

### Efficacy

#### Abatacept retention

**Fig. 2:**
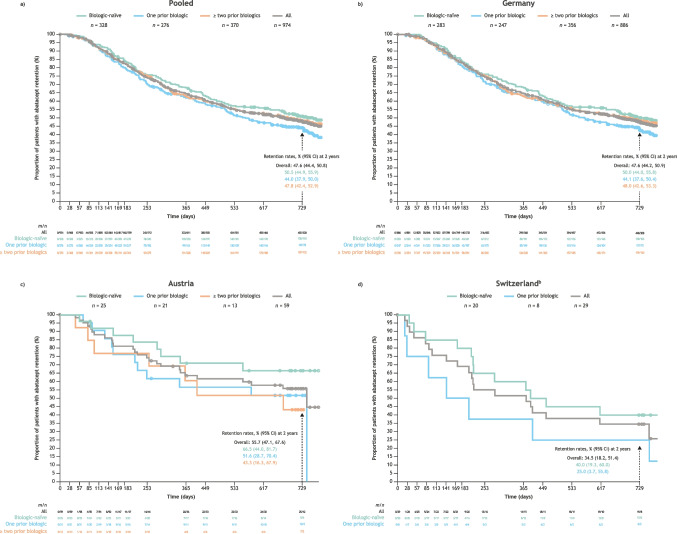
Proportion of patients with subcutaneous abatacept retention over 2 years by treatment line^a^, for **a**) the pooled cohort, **b**) Germany, **c**) Austria, and **d**) Switzerland. ^a^Patients who switched to IV abatacept during the 2 years were discontinued and are not included here. ^b^Data for Switzerland ≥ two prior biologics treatment group not presented to maintain confidentiality due to small patient population. *CI*, confidence interval; *IV*, intravenous; *m*, number of patients censored; *n*, number of patients at risk

For the pooled cohort, the abatacept retention rate (95% CI) at 2 years was 47.6% (44.4, 50.8) (Fig. 2a), with the highest rate in patients who were biologic-naïve (50.5% [44.9, 55.9]) and the lowest rate in patients with one prior biologic (44.0% [37.9, 50.0]). In the Germany cohort, similar to the pooled cohort, the highest retention was seen in patients who were biologic-naïve and the lowest retention was seen in patients with one prior biologic (Fig. 2b). In the Austria cohort, the highest retention was observed in patients who were biologic-naïve and the lowest retention was observed in patients with ≥ two prior biologics (Fig. 2c). In the Switzerland cohort, retention was also highest in biologic-naïve patients (Fig. 2d).

In the pooled cohort and across treatment line groups, lack of efficacy (*n* = 235/507 [46.4%]) was the most common reason for discontinuation of abatacept. Intolerance/safety issues (*n* = 117/507 [23.1%]) was the second most common reason for discontinuation, followed by “other” reasons (*n* = 87/507 [17.2%]). Similar to the pooled cohort, the Germany, Austria, and Switzerland cohorts reported lack of efficacy (*n* = 215/464 [46.3%], *n* = 9/23 [39.1%], and *n* = 11/20 [55.0%], respectively) as the most common reason for discontinuing abatacept.

**Fig. 3:**
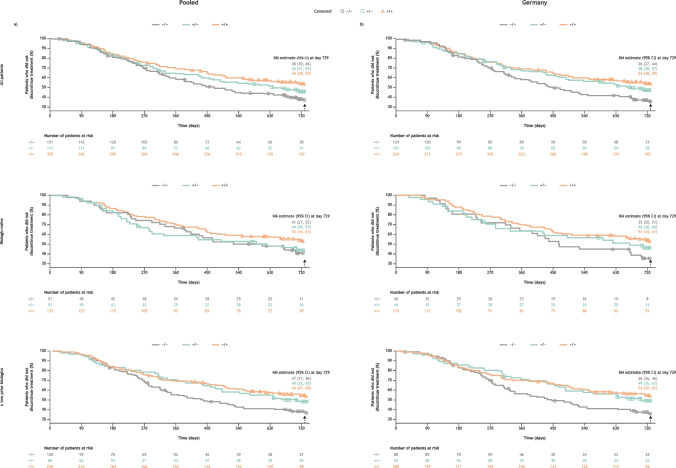
Proportion of patients with subcutaneous abatacept retention over 2 years by baseline RF/ACPA serostatus and treatment line, for **a**) the pooled cohort, and **b**) Germany**.** Patients with missing data for baseline RF/ACPA status are excluded. Patients with time to discontinuation greater than 2 years are censored at day 729. Data for Austria and Switzerland are not presented due to low patient numbers. + */* + , ACPA + and RF + ; + */ − *, ACPA + or RF + ; − */ − *, ACPA − and RF − ; *ACPA*, anti-citrullinated protein antibody; *CI*, confidence interval; *KM*, Kaplan–Meier; *RF*, rheumatoid factor

For both the pooled cohort and the Germany cohort, patients who were + / + at baseline had higher retention of abatacept over 2 years than patients who were + / − or − / − , irrespective of treatment line (Fig. 3). For the pooled cohort, retention rate (95% CI) was 54% (48, 59) for patients who were + / + at baseline, 46% (37, 55) for patients who were + / − , and 38% (30, 46) for patients who were − / − (Fig. 3a). For the German cohort, retention rate (95% CI) was 54% (48, 59) for patients who were + / + at baseline, 48% (38, 57) for patients who were + / − , and 36% (27, 44) for patients who were − / − (Fig. 3b).

In the pooled cohort analysis, regardless of treatment line, a significantly lower proportion of patients who were + / + discontinued abatacept treatment than patients who were − / − (*p* = 0.015; Supplementary Fig. 1). For all patients in the pooled cohort and for patients who were biologic-naïve, the adjusted risk of discontinuation was 30% less for patients who were + / + than patients who were − / − . Patients with ≥ two prior biologics who were + / + or + / − were 40% and 30% less likely, respectively, to discontinue abatacept than patients who were − / − . In the Germany cohort, a significantly lower proportion of patients who were + / + discontinued abatacept treatment than patients who were − / − , regardless of prior treatment status (*p* = 0.008; Supplementary Fig. 1). For all patients in the Germany cohort and for patients who were biologic-naïve in the Germany cohort, the adjusted risk of discontinuation was 40% less for patients who were + / + than patients who were − / − and 20% less for patients who were + / − than patients who were − / − .

#### Clinical outcomes

**Fig. 4:**
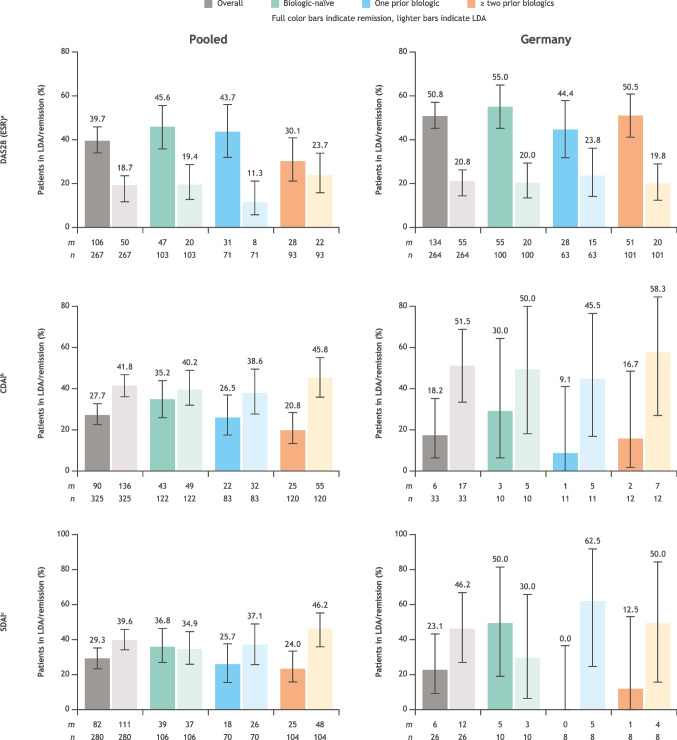
Clinical outcomes with subcutaneous abatacept over 2 years. ^a^Remission < 2.6; LDA ≥ 2.6 to ≤ 3.2. ^b^Remission ≤ 2.8; LDA ≤ 10. ^c^Remission ≤ 3.3; LDA ≤ 11. *CDAI*, Clinical Disease Activity Index; *DAS28 (ESR)*, Disease Activity Score in 28 joints based on the erythrocyte sedimentation rate; *LDA*, low disease activity; *m*, number of patients in LDA or remission; *n*, number of patients with complete data at 2 years; *SDAI*, Simplified Disease Activity Index

In the pooled cohort, at 2 years, a higher proportion of patients who were biologic-naïve were in DAS28 (ESR), CDAI, or SDAI remission compared with patients with one or ≥ two prior biologics (Fig. 4). A higher proportion of patients with ≥ two prior biologics had DAS28 (ESR), CDAI, or SDAI LDA than patients who were biologic-naïve or had one prior biologic. Trends in the Germany cohort generally followed the same pattern as those in the pooled cohort: the proportion of patients in DAS28 (ESR), CDAI, or SDAI remission was highest among patients who were biologic-naïve (Fig. 4). Generally, the proportion of patients with LDA was highest in the ≥ two prior biologics group.

### Safety

Safety profiles were similar across country cohorts and treatment lines (Table 2). No new safety signals for SC abatacept were identified. In the pooled cohort, 573 patients (58.4%) had at least one AE and 169 patients (17.2%) had at least one SAE.

**Table 2:** Summary of adverse events

	Pooled				Germany				Austria				Switzerland			
	All, *N* = 981	Biologic-naïve, *n* = 332	One prior biologic, *n* = 279	≥ two prior biologics, *n* = 370	All, *n* = 890	Biologic-naïve, *n* = 286	One prior biologic, *n* = 248	≥ two prior biologics, *n* = 356	All, *n* = 61	Biologic-naïve, *n* = 26	One prior biologic, *n* = 22	≥ two prior biologics, *n* = 13	All, *n* = 30	Biologic-naïve, *n* = 20	One prior biologic, *n* = 9	≥ two prior biologics, *n* = 1^a^
At least one adverse event	573 (58.4)	197 (59.3)	163 (58.4)	213 (57.6)	530 (60.0)	173 (60.5)	149 (60.1)	208 (58.4)	28 (45.9)	14 (53.8)	10 (45.5)	4 (30.8)	15 (50.0)	10 (50.0)	4 (44.4)	NP
At least one serious adverse event	169 (17.2)	59 (17.8)	50 (17.9)	60 (16.2)	154 (17.3)	51 (17.8)	45 (18.1)	58 (16.3)	11 (18.0)	6 (23.1)	3 (13.6)	2 (15.4)	4 (13.3)	2 (10.0)	2 (22.2)	NP
At least one related adverse event	264 (26.9)	79 (23.8)	77 (27.6)	108 (29.2)	242 (27.2)	67 (23.4)	70 (28.2)	105 (29.5)	15 (24.6)	6 (23.1)	6 (27.3)	3 (23.1)	7 (23.3)	6 (30.0)	1 (11.1)	NP
Deaths^b^	8 (1.6) *n* = 507	4 (2.5) *n* = 162	3 (2.0) *n* = 153	1 (0.5) *n* = 192	8 (1.7) *n* = 464	4 (2.8) *n* = 143	3 (2.2) *n* = 137	1 (0.5) *n* = 184	0 (0.0)	0 (0.0)	0 (0.0)	0 (0.0)	0 (0.0)	0 (0.0)	0 (0.0)	NP

Data are shown as n (%)

^a^Data for Switzerland ≥ two prior biologics treatment group are not presented to maintain confidentiality due to small patient population (*n* = 1)

^b^Death as a reason for discontinuation of abatacept

*NP*, not presented

## Discussion

In this post hoc analysis of the real-world ASCORE study including patients from Germany, Austria, and Switzerland, 47.6% of patients were retained on abatacept 2 years after treatment initiation. Serostatus at baseline appeared to influence retention: for both the pooled and Germany cohorts, patients who were + / + demonstrated higher retention of abatacept than patients who were − / − or + / − over 2 years, irrespective of treatment line. With regard to treatment lines, retention of abatacept was highest in patients who were biologic-naïve at baseline compared with patients with one or ≥ two prior biologic treatments. Additionally, at 2 years, a higher proportion of patients who were biologic-naïve were in clinical remission (as assessed with DAS28 [ESR], CDAI, and SDAI) compared with patients who had previously been treated with a bDMARD.

Similar to the current analysis, the overall retention rate of SC abatacept was 47.3% in the global ASCORE population [17]. Further in line with this finding, the overall retention rate of IV abatacept was 48% in the AbataCepT In rOutiNe clinical practice (ACTION) study (2008–2013) [16]. Other studies have shown a range of abatacept retention rates from 39 to 83%, depending on the patient population, route of administration (IV or SC), follow-up time, and study location [21–23].

Our finding that abatacept retention was higher among patients who were biologic-naïve (50.5%) compared with those with one or ≥ two prior biologics (44.0% and 47.8%, respectively) is aligned with the global ASCORE data, which showed the highest retention rate of 51.7% in biologic-naïve patients (compared with one or ≥ two prior biologics, 45.6% and 43.2%, respectively) [17]. This finding is also consistent with those from other previous European studies [24, 25]. Similarly, retention rates reported in the ACTION study for earlier lines of IV abatacept treatment were higher than those for later lines of treatment in patients with RA (63% for biologic-naïve and 47% for biologic-failure patients) [26, 27]. Together with our analysis, these findings support the early versus later-line use of abatacept treatment in patients with RA.

Consistent with previous studies [23, 28], here we report that retention of abatacept was higher among patients from Germany, Austria, and Switzerland who were + / + at baseline compared with patients who were − / − (54% versus 38% for all patients pooled). Similar observations demonstrating higher retention rates in patients who were + / + compared with those who were − / − have been reported from the global ASCORE study (57% versus 37% for biologic-naïve patients) [17]. In the present analysis, patients with + / + RA had a 30% lower likelihood of discontinuation of abatacept than patients with − / − RA. A similar observation was noted in the ACTION study for patients who were biologic-naïve (29% lower chance for discontinuation of abatacept for patients with + / + versus − / − RA) [16]. Of clinical relevance, the exploratory analyses of the ASCORE study by serostatus showed a greater reduction in disease activity (SDAI and CDAI) for patients who were + / + treated with first-line abatacept than patients with RA receiving later-line therapy and patients who were − / − [17]. Furthermore, a higher proportion of patients who were + / + achieved SDAI and CDAI LDA and/or remission compared with patients who were − / − [17]. Altogether, those who are + / + show higher retention of abatacept treatment and better clinical outcomes after abatacept treatment, than patients who are − / − . The mechanism of action of abatacept [29], or the homogeneity of patients with seropositive RA, may help explain the improved retention and clinical responses but a precise understanding remains unclear. Nevertheless, given the development of precision medicine, seropositivity among patients with RA may provide a key prognostic screening factor capable of guiding treatment prescription [17].

Interim analyses of the ASCORE study at 6 months and 1 year showed better clinical response rates in patients receiving SC abatacept as a first-line versus later-line bDMARD [18, 19]. Similarly, other previous studies have shown patients who are biologic-naïve are more likely to experience a greater clinical response with abatacept than patients who previously failed ≥ 1 biologic treatment [17, 25]. Among the treatment lines in this analysis, the proportion of patients who achieved DAS28 (ESR), SDAI, and CDAI remission at 2 years was highest in the biologic-naïve cohort compared with patients with 1 or ≥ 2 prior biologics. While we reported retention rates by treatment line and country, clinical outcomes were only reported for Germany and all countries pooled owing to small sample sizes in the Austria and Switzerland cohorts. Overall, abatacept was well tolerated over 2 years in this analysis, with no new safety signals, consistent with the results of previous analyses [17, 24, 30].

This analysis had several strengths, the first of which being the site selection process. The rheumatologists involved in the study were randomly selected from country-specific nationwide independent databases of specialists located in hospitals or private practice for a well-balanced geographic distribution and were representative of specialists caring for patients with RA in each participating country [17]. Of note, the included German-speaking countries comprised one-third of the global ASCORE study, and in turn, represented the largest geographic subgroup. Importantly, the further breakdown of the global study, as reported here, provides important data and guidance for physicians in the respective countries. As with the global ASCORE study, patients were stratified by treatment line to assess the effect of different stages of treatment on abatacept efficacy and retention. Serostatus is currently being investigated with increasing vigour as a factor that may enable physicians to further adapt treatment strategies for specific patient groups. Therefore, in this analysis, patients were also stratified by serostatus to assess the effect on abatacept retention and to provide clinicians with real-world evidence that may be applied to their clinical settings.

This analysis had three main limitations. First, as a breakout analysis of a global study and although relevant to local clinicians, the patient sample in this analysis may not be representative of patients with RA in other countries. Second, as this was an observational study, there was potential referral and channelling bias, no active comparator, and loss of patients to follow-up. Of note, only 2.9% of patients were lost to follow-up over 2 years in the ASCORE study, as the study design did not interfere with usual clinical practice [17]. Third, the sample sizes of the Switzerland and Austria cohorts were very small when stratified by treatment line and serostatus, which may have undermined the validity of the results in these cohorts.

In this post hoc analysis of the ASCORE study of patients from Germany, Austria, and Switzerland, abatacept demonstrated a retention rate of 47.6% and good clinical outcomes after 2 years. Retention of abatacept was higher in patients who were biologic-naïve than in patients with one or ≥ two prior biologics at baseline and in those with either + / + or + / − RA. Retention of abatacept was also higher in patients with + / + RA when compared with patients with − / − RA. No new safety signals were identified in this analysis, and there was little variation among SAEs between countries. Our findings demonstrate that initiating abatacept as an early versus later-line treatment may provide better disease control and clinical outcomes in patients with RA. Furthermore, seropositivity may guide precision medicine efforts by providing a prognostic factor capable of identifying patients able to demonstrate a higher abatacept treatment response. These real-world data may be useful for clinicians to inform individualized treatment pathways for patients with RA, fostering superior disease control and clinical outcomes.

## Supplementary information

Below is the link to the electronic supplementary material.

Supplementary file1 (PDF 240 KB)

## Data Availability

The data that support the findings of this study are available from Bristol Myers Squibb but restrictions apply to the availability of these data, which were used under license for the current study, and so are not publicly available. Data are available from the authors upon reasonable request and with permission of Bristol Myers Squibb. Data requests are sent through an independent review committee to evaluate who provide the final decision on requests. Bristol Myers Squibb policy on data sharing may be found at https://www.bms.com/researchers-and-partners/independent-research/data-sharing-request-process.html. All authors had full access to all of the primary data in this study and agree to allow the journal to review their data if requested.
