# Supplementary material for: Subcutaneous abatacept for the treatment of rheumatoid arthritis in routine clinical practice in Germany, Austria, and Switzerland: 2-year retention and efficacy by treatment line and serostatus
Source: Clin Rheumatol. 2023 Jun 14;42(9):2321–34. doi: 10.1007/s10067-023-06649-x (PMC10412468; doi:10.1007/s10067-023-06649-x)
Supplement: Supplementary file 1 — Supplementary file1 (PDF 240 KB) [file 10067_2023_6649_MOESM1_ESM.pdf]

Supplementary Information

Subcutaneous abatacept for the treatment of rheumatoid arthritis in routine clinical practice in Germany, Austria, and Switzerland: two-year retention and efficacy by treatment line and serostatus

Rieke Alten[1] • Hans-Peter Tony[2] • Bettina Bannert[3] • Hubert Nüßlein[4] • Christiane Rauch[5,a] • Sean E. Connolly[6] • Melanie Chartier[7] • Karissa Lozenski[6] • Roland Hackl[8] • Adrian Forster[9] • Peter Peichl[10]

[1]Department of Internal Medicine, Rheumatology, Schlosspark-Klinik, University Medicine Berlin, Heubnerweg 2, Berlin 14059, Germany
[2]Medizinische Klinik und Poliklinik II, Rheumatologie/Klinische Immunologie, Universitätsklinikum Würzburg, Josef-Schneider-Straße 2, Würzburg 97080, Germany
[3]Rheumatologische Universitätsklinik, Universitätsspital Basel, Petersgraben 4, Basel 4031, Switzerland
[4]Medic-Center Nürnberg (private practice), Gibitzenhofstraße 150, 90443 Nürnberg, Germany
[5]Medical Immunology & Fibrosis, Bristol Myers Squibb, Arnulfstraße 29, Munich 80636, Germany
[6]Immunology and Fibrosis/Global Drug Development, Bristol Myers Squibb, 3401 Princeton Pike, Lawrenceville, NJ 08540, USA
[7]MESP France – Market Access, Bristol Myers Squibb, 3 Rue Joseph Monier, Rueil-Malmaison 92506, France
[8]Immuno-Oncology, Bristol Myers Squibb, Handelskai 92/Rivergate/Gate 1, 5. OG, Vienna 1200, Austria
[9]Department of Rheumatology, Schulthess Klinik, Lengghalde 2, Zürich 8008, Switzerland
[10]Department of Internal Medicine, Evangelical Hospital, Hans-Sachs-Gasse 10-12, Vienna 1180, Austria

Corresponding author: Rieke Alten; email: Rieke.Alten@schlosspark-klinik.de

[a]At the time of analysis

Journal: *Clinical Rheumatology*

Contents

- **Supplementary Table 1** Patient demographics and baseline disease characteristics by treatment line and baseline serostatus for the pooled analysis cohort
- **Supplementary Table 2** Patient demographics and baseline disease characteristics by treatment line and baseline serostatus for Germany
- **Supplementary Fig. 1** Adjusted risk of discontinuation of abatacept for patients stratified by treatment line and baseline serostatus

Supplementary Table 1 Patient demographics and baseline disease characteristics by treatment line and baseline serostatus for the pooled analysis cohort

	**All patients**			**Biologic-naïve**			**≥ two prior biologics**		
**Characteristic**	**+/+** ***n* = 362**	**+/−** ***n* = 118**	**−/−** ***n* = 152**	**+/+** ***n* = 133**	**+/−** ***n* = 52**	**−/−** ***n* = 52**	**+/+** ***n* = 229**	**+/−** ***n* = 66**	**−/−** ***n* = 100**
Age, years	58.5 (12.2)	58.1 (12.5)	57.8 (14.4)	59.1 (12.9)	56.7 (12.9)	59.2 (16.3)	58.2 (11.8)	59.2 (12.2)	57.1 (13.3)
BMI, kg/m^2	27.2 (5.4)	27.3 (5.4)	28.1 (5.8)	26.9 (5.5)	27.0 (5.1)	27.9 (5.3)	27.3 (5.4)	27.4 (5.6)	28.2 (6.0)
ACPA positive, *n* (%)	362 (100.0)	80 (67.8)	0 (0.0)	133 (100.0)	32 (61.5)	0 (0.0)	229 (100.0)	48 (72.7)	0 (0.0)
RF positive, *n* (%)	362 (100.0)	38 (32.2)	0 (0.0)	133 (100.0)	20 (38.5)	0 (0.0)	229 (100.0)	18 (27.3)	0 (0.0)
DAS28 (ESR)	5.0 (1.3)	4.8 (1.2)	5.0 (1.4)	5.0 (1.3)	4.8 (1.0)	5.1 (1.4)	5.0 (1.3)	4.9 (1.4)	4.9 (1.4)
DAS28 (CRP)	4.7 (1.1)	4.4 (1.1)	4.6 (1.2)	4.7 (1.2)	4.4 (1.0)	4.6 (1.2)	4.7 (1.1)	4.4 (1.2)	4.6 (1.3)
CDAI	25.6 (12.3)	23.7 (11.4)	26.8 (13.1)	25.1 (11.7)	23.0 (12.1)	25.8 (11.6)	25.9 (12.7)	24.2 (10.9)	27.4 (14.0)
SDAI	27.1 (12.5)	24.8 (11.4)	27.9 (13.5)	26.5 (12.0)	23.8 (11.6)	26.8 (11.4)	27.4 (12.8)	25.6 (11.4)	28.5 (14.4)

Data are shown as mean (SD) unless otherwise specified.
+/+, ACPA/RF double positive; *+/−*, ACPA or RF single positive; *−/−*, ACPA/RF double negative; *ACPA*, anti-citrullinated protein antibody; *BMI*, body mass index; *CDAI*, Clinical Disease Activity Index; *CRP*, C-reactive protein; *DAS28*, Disease Activity Score in 28 joints; *ESR*, erythrocyte sedimentation rate; *RF*, rheumatoid factor; *SD*, standard deviation; *SDAI*, Simplified Disease Activity Index.

Supplementary Table 2 Patient demographics and baseline disease characteristics by treatment line and baseline serostatus for Germany

	**All patients**			**Biologic-naïve**			**≥ two prior biologics**		
**Characteristic**	**+/+ *n* = 325**	**+/− *n* = 107**	**−/− *n* = 125**	**+/+ *n* = 116**	**+/− *n* = 44**	**−/− *n* = 37**	**+/+ *n* = 209**	**+/− *n* = 63**	**−/− *n* = 88**
Age, years	59.1 (11.8)	57.6 (12.5)	57.5 (14.4)	59.8 (12.4)	56.2 (13.2)	57.9 (17.0)	58.8 (11.5)	58.6 (12.1)	57.4 (13.2)
BMI, kg/m^2	27.4 (5.4)	27.5 (5.3)	28.2 (5.7)	27.2 (5.5)	27.7 (4.9)	27.5 (5.3)	27.4 (5.3)	27.3 (5.6)	28.4 (5.9)
ACPA positive, *n* (%)	325 (100.0)	75 (70.1)	0 (0.0)	116 (100.0)	29 (65.9)	0 (0.0)	209 (100.0)	46 (73.0)	0 (0.0)
RF positive, *n* (%)	325 (100.0)	32 (29.9)	0 (0.0)	116 (100.0)	15 (34.1)	0 (0.0)	209 (100.0)	17 (27.0)	0 (0.0)
DAS28 (ESR)	5.0 (1.3)	4.9 (1.2)	5.0 (1.4)	5.0 (1.3)	5.0 (0.9)	5.1 (1.3)	5.0 (1.3)	4.8 (1.4)	4.9 (1.4)
DAS28 (CRP)	4.7 (1.2)	4.5 (1.1)	4.6 (1.3)	4.7 (1.2)	4.7 (0.9)	4.5 (1.2)	4.6 (1.2)	4.4 (1.2)	4.6 (1.3)
CDAI	25.4 (12.6)	24.2 (11.7)	26.8 (13.9)	25.2 (12.1)	25.0 (12.8)	25.1 (12.0)	25.6 (12.9)	23.7 (10.9)	27.4 (14.6)
SDAI	27.0 (12.9)	25.5 (11.6)	27.8 (13.9)	26.8 (12.5)	26.2 (12.2)	26.3 (10.8)	27.1 (13.2)	25.0 (11.3)	28.4 (14.9)

Data are shown as mean (SD) unless otherwise specified.
+/+, ACPA/RF double positive; +/−, ACPA or RF single positive; −/−, ACPA/RF double negative; *ACPA*, anti-citrullinated protein antibody; *BMI*, body mass index; *CDAI*, Clinical Disease Activity Index; *CRP*, C-reactive protein; *DAS28*, Disease Activity Score in 28 joints; *ESR*, erythrocyte sedimentation rate; *RF*, rheumatoid factor; *SD*, standard deviation; *SDAI*, Simplified Disease Activity Index.

Supplementary Fig. 1 Adjusted risk of discontinuation of abatacept for patients stratified by treatment line and baseline serostatus

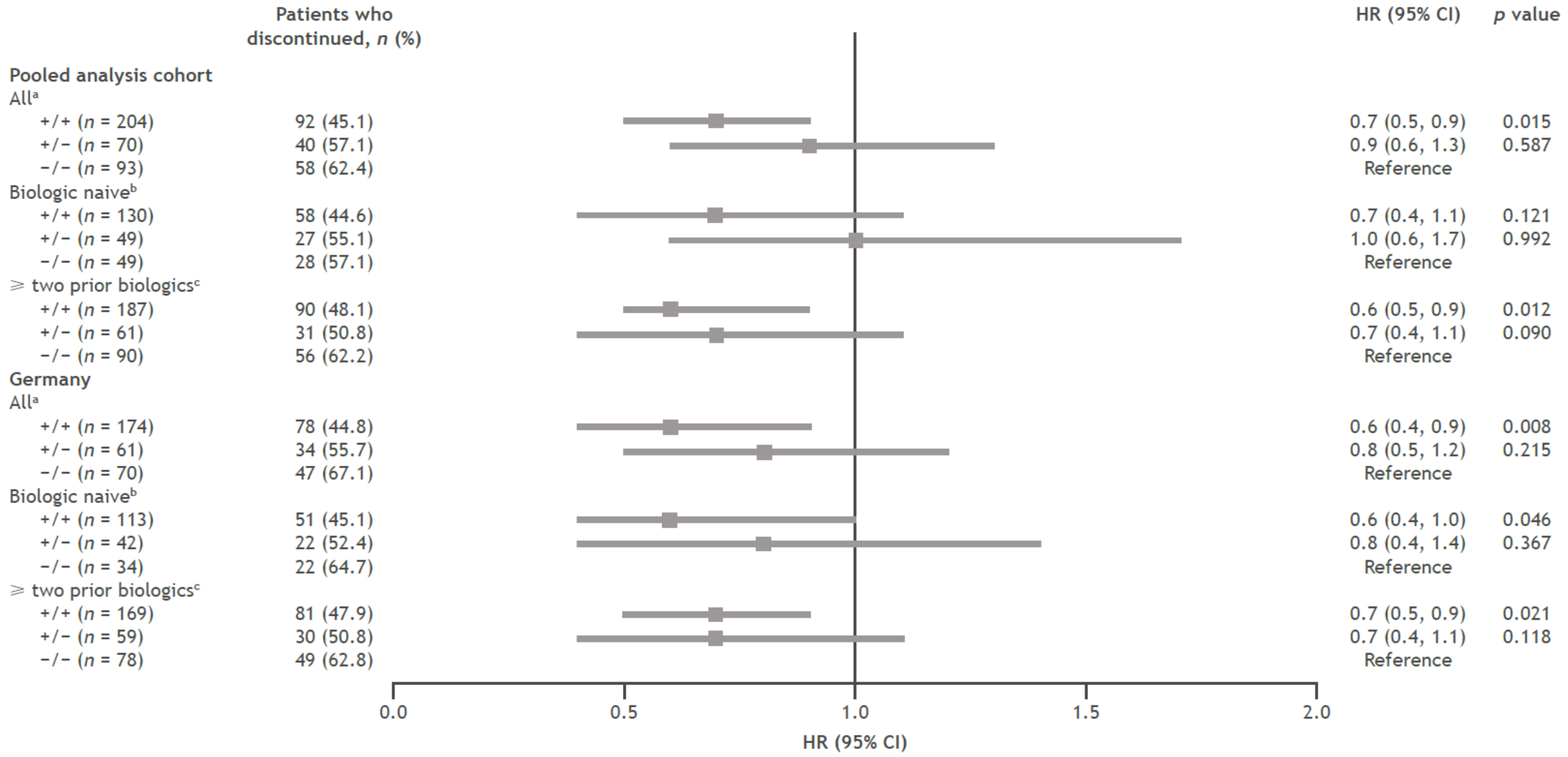

Data obtained from a Cox proportional hazards model.
[a]Adjusted for RF/ACPA status, employment status, ESR (mm), severity of pain, treatment pattern at abatacept initiation (2 levels), and ≥ 1 prior biologic.

[b]Adjusted for RF/ACPA status and employment status.
[c]Adjusted for RF/ACPA status, ESR (mm), and treatment pattern at abatacept initiation (two levels).
+/+, ACPA/RF double positive; +/−, ACPA or RF single positive; −/−, ACPA/RF double negative; *ACPA*, anti-citrullinated protein antibody; *CI*, confidence interval; *ESR*, erythrocyte sedimentation rate; *HR*, hazard ratio; *RF*, rheumatoid factor.
